# Nb_2_C MXene-Functionalized Scaffolds Enables Osteosarcoma Phototherapy and Angiogenesis/Osteogenesis of Bone Defects

**DOI:** 10.1007/s40820-020-00547-6

**Published:** 2021-01-04

**Authors:** Junhui Yin, Shanshan Pan, Xiang Guo, Youshui Gao, Daoyu Zhu, Qianhao Yang, Junjie Gao, Changqing Zhang, Yu Chen

**Affiliations:** 1 Institute of Microsurgery On Extremities, Shanghai Jiao Tong University Affiliated Sixth People’s Hospital, Shanghai, 200233 People’s Republic of China; 2 School of Life Sciences, Shanghai University, Shanghai, 200444 People’s Republic of China; 3 State Key Laboratory of High Performance Ceramics and Superfine Microstructure, Shanghai Institute of Ceramics, Chinese Academy of Sciences, Shanghai, 200050 People’s Republic of China; 4 Department of Orthopaedic Surgery, Shanghai Jiao Tong University Affiliated Sixth People’s Hospital, Shanghai, 200233 People’s Republic of China; 5 Department of Orthopedics, The Second Affiliated Hospital, The Navy Medical University, Shanghai, 200003 People’s Republic of China

**Keywords:** Nb_2_C MXene, 3D printing, Phototherapy, Osteosarcoma, Vascularization

## Abstract

Early surgical resection and chemotherapy of bone cancer are commonly used in the treatment of bone tumor, but it is still highly challenging to prevent recurrence and fill the bone defect caused by the resection site. In this work, we report a rational integration of photonic-responsive two-dimensional (2D) ultrathin niobium carbide (Nb_2_C) MXene nanosheets (NSs) into the 3D-printed bone-mimetic scaffolds (NBGS) for osteosarcoma treatment. The integrated 2D Nb_2_C-MXene NSs feature specific photonic response in the second near-infrared (NIR-II) biowindow with high tissue-penetrating depth, making it highly efficient in killing bone cancer cells. Importantly, Nb-based species released by the biodegradation of Nb_2_C MXene can obviously promote the neogenesis and migration of blood vessels in the defect site, which can transport more oxygen, vitamins and energy around the bone defect for the reparative process, and gather more immune cells around the defect site to accelerate the degradation of NBGS. The degradation of NBGS provides sufficient space for the bone remodeling. Besides, calcium and phosphate released during the degradation of the scaffold can promote the mineralization of new bone tissue. The intrinsic multifunctionality of killing bone tumor cell and promoting angiogenesis and bone regeneration makes the engineered Nb_2_C MXene-integrated composite scaffolds a distinctive implanting biomaterial on the efficient treatment of bone tumor.

**Electronic supplementary material:**

The online version of this article (10.1007/s40820-020-00547-6) contains supplementary material, which is available to authorized users.

## Highlights

- 2D Nb_2_C MXene-integrated 3D-printing scaffolds against osteosarcoma were constructed with theragenerative functionality.
- Nb_2_C MXene in 3D scaffolds enabled photothermal ablation of osteosarcoma at NIR-II biowindow.
- Nb_2_C MXene in 3D scaffolds promoted osteogenesis, osteoconduction and osteoinduction, and drove vascularization for bone regeneration.

**Electronic supplementary material:**

The online version of this article (10.1007/s40820-020-00547-6) contains supplementary material, which is available to authorized users.

## Graphical abstract

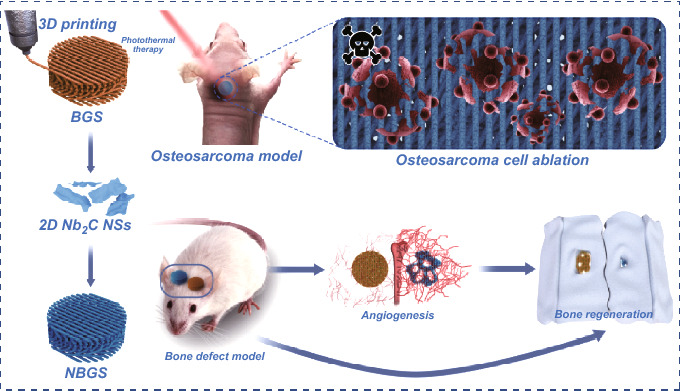

## Introduction

Bone tumor is one of the most common malignancies among children and adolescents, with poor long-term survival rate [1–3]. Currently, surgical resection is the prevalent strategy to treat bone tumor [4]. However, the invasive nature of bone malignancies determines remnant tumor cell, which might induce local recurrence and surgical failure. Meanwhile, bone defect secondary to tumor resection is largely critical and skeletal repair is essential to anatomical reconstruction and functional recovery [4, 5]. Although a substantial number of bone defects can be cured using the gold standard technique of autologous bone grafting, the donor site is limited, and potential complications are the rising concerns following bone harvest [6, 7]. Various bone defects contribute to heavy healthy and socioeconomic burdens worldwide [8].

The emerging bone tissue engineering and precision medicine have shed light on curing critical bone defect and tumor-targeted therapy in the past decade. On the one hand, biomaterials with high osteoconductivity, osteoinductivity and osseointegration are promising to repair bone defects, but few of them are qualified in tumor ablation [9, 10]. On the other hand, the drug-loading and controlled-release system, especially the nanomaterials, can substantially improve the efficiency of targeted therapy of bone tumor [11], but their therapeutic role may be effected by drug resistance after continuous chemotherapy. Multifunctional materials with tumor ablation and bone-remodeling capacities are expected to be developed for clinical bone tumor treatment [12–15].

Two-dimensional (2D) MXene nanosheets (NSs) have emerged as the representative 2D layer structured materials comprised by carbides, nitrides or carbonitrides with abundant unique physiochemical properties [16]. In particular, 2D niobium carbide (Nb_2_C) MXene NSs are highly biocompatible and biodegradable with intrinsic photoresponse in the second near-infrared (NIR-II) biological window for theranostic nanomedicine [17, 18]. Very recently, we have demonstrated that Nb_2_C NSs acted as the photothermal conversion nanoagents for near-infrared (NIR)-triggered photonic hypothermia against breast cancer after intravenous administration [16], making them highly applicable in the potential treatment of osteosarcoma. However, their therapeutic efficiency and biological effect in the sequential processes of photonic osteosarcoma ablation in the NIR-II biowindow and biomaterial-guided bone regeneration have not been achieved, which require further investigations for promoting 2D Nb_2_C MXene into tissue regeneration biomedical field. Three-dimensional (3D)-printed bioactive glass scaffolds (BGS) are featured with favorable degradation rate [19], stable drug release [20], high biocompatibility [21] and satisfactory osteoconductivity/osteoinductivity [22]. Therefore, the rational integration of 2D Nb_2_C MXenes into 3D porous BGS (NBGS) is expected to construct a multifunctional scaffold with specific functionality of photonic bone tumor hyperthermia and improved bone regeneration.

**Scheme 1:**
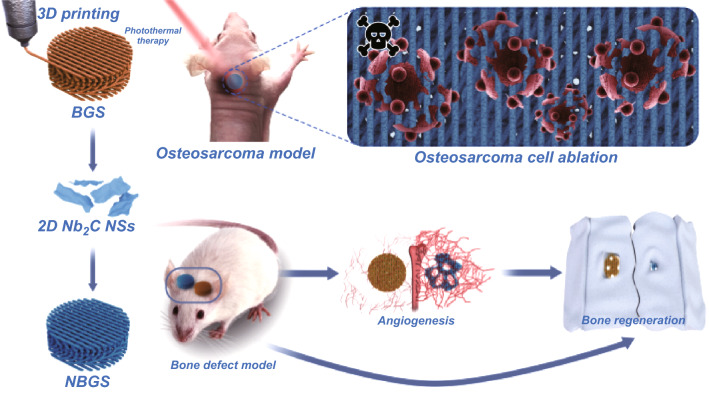
Schematic diagram of the process for photothermal ablation of osteosarcoma and bone regeneration by NBGS. Vascularization can be promoted to enhance osseous reconstruction

Herein, the ultrathin Nb_2_C MXene NSs were integrated into a 3D-printed bone-mimetic BGS, for vitalizing the composite scaffolds with specific capability of photonic bone malignancy ablation in the NIR-II biowindow, while driving osseous regeneration by promoted neovascularization (Scheme 1). Noteworthy, the constructed NBGS are highly preferable for osteosarcoma treatment. Initially, the photothermal hyperthermia was conducted after the implantation of multifunctional NBGS to kill bone tumor cells. Subsequently, the significant vascularization emerged to drive new osseous formation, and the coupled formation of blood vessels and bone structures were beneficial for the rapid repair of large bone defect following the gradual degradation of the scaffolds. Therefore, the constructed multifunctional NBGS provides a distinctive biomaterial scaffold for bone tumor treatment with simultaneous tumor therapeutic and bone tissue regeneration capabilities.

## Experimental Section

### Synthesis of NBGS

The bioactive glass scaffolds (BGS) with bone-mimetic structure and the 2D Nb_2_C NSs were, respectively, prepared according to our previous reports [12, 15]. Subsequently, the BGS were soaked in Nb_2_C NSs aqueous solution (10 min, at room temperature) and then put in the drier (37 °C, 4 h). The above steps were repeated three times, and the Nb_2_C NS-modified BGS (NBGS) were obtained. BGS integrated with 0.5 mg mL^−1^ Nb_2_C NSs was denominated as 0.5 NBGS, and the other NBGS were also termed by this analogy.

### Characterization

Morphological images, energy-dispersive X-ray spectroscopy (EDS) and corresponding elemental mapping were obtained from a scanning electron microscopy (SEM, field emission Magellan 400). Transmission electron microscopy (TEM) images were recorded by a transmission electron microscope (JEM-2100F transmission electron microscope, 200 kV). The elemental banding energy was analyzed by X-ray photoelectron spectroscopy (XPS, ESCAlab250, Thermo Scientific). Raman spectra of biomaterials were acquired by a Raman microscope (HORIBA LabRAM HR800). Phase analysis was calculated by X-ray diffractometer (XRD, Rigaku D/MAX-2200PC). Mineralization function of the scaffolds was assessed in simulated body fluid (SBF). BGS and NBGS were soaked in SBF for 24 h at 37 °C. Then they were dried at 60 °C for subsequent analysis of surface morphology and element with a SEM–EDS system.

### In Vitro Photothermal Effect of NBGS

BGS, 0.25 NBGS, 0.5 NBGS and 1.0 NBGS were irradiated by 1064-nm laser, and corresponding temperature curves and thermal images were obtained by an infrared thermal imaging camera (PLIR A325SC camera, USA). 1.0 NBGS were further exposed to laser irradiation at different power densities of 0.25, 0.5, 0.75 and 1.0 W cm^−2^, 5 min, in phosphate-buffered saline (PBS).

### In Vitro Cytotoxicity and Tumor cell Ablation Assays

Saos-2 cells (human osteosarcoma tumor cells) were cultured with Dulbecco's modified Eagle media (DMEM) supplemented with 1% penicillin/streptomycin and 10% fetal bovine serum (FBS). For in vitro cytotoxicity and cell ablation assay in NIR-II biowindow, Saos-2 cells were inoculated into 48-well plates (1.0 × 10^5^ cells per well) for 12 h to ensure cellular attachment; then, BGS and 1.0 NBGS were cultured with Saos-2 cells for additional 24 h, respectively. According to the scheme, these samples were reasonably divided into six groups (blank, laser only, BGS, BGS + laser, NBGS, NBGS + laser; all lasers with 1064 nm wavelength irradiated at 1.0 W cm^−2^ power density for 5 min; n = 6). In order to investigate the effect of 1064-nm laser irradiation on tumor cell ablation, irradiation durations (0, 5, 10 and 15 min), irradiation cycles (0, 1, 2 and 3 times) and power densities (0, 0.5, 0.75 and 1.0 W cm^−2^) of photobased thermal ablation were further systematically evaluated. Eventually, the OD value was detected by the Cell Counting Kit-8 (CCK-8) assay after various treatments.

### Live/dead Fluorescence Observation

Saos-2 cells were seeded into culture dishes (1.0 × 10^5^ cells per dish; NEST Biotechnology Co. LTD, Hong Kong, China) for 24 h. Propidium iodide (PI) and calcein AM were added to the culture medium with Saos-2 cells from different groups (BGS, BGS + 1064-nm laser, NBGS, NBGS + 1064-nm laser; all lasers were operated at 1.0 W cm^−2^ power density for 5 min) and the cell status was observed by a confocal laser scanning microscopy (CLSM, Olympus BX53, Olympus, Japan).

### Vasculogenesis Assay

The effect of BGS/NBGS on the capacity of cell migration and tube formation was assessed using human umbilical vein endothelial cells (HUVECs), which were originally purchased from Cell Bank of Shanghai Institutes for Biological Sciences, Chinese Academy of Sciences, Shanghai, China. Cells were cultured with endothelial cell medium (ECM) (ScienCell, Carlsbad, CA, USA). For wound healing assay, cells were seeded in a Culture-Insert (ibidi GmbH, Gräfelfing, Germany). Four hours later, a cell-free gap was made, and cells were co-cultured with BGS/NBGS for 24 h to measure the migration of HUVECs. For transwell migration assay, cells were seeded into the upper chamber of 12-well transwell plates, while BGS/NBGS were placed in the lower chamber with complete ECM plus 10% FBS. After 24 h, HUVECs were stained with 0.5% crystal violet and the migrated cells were imaged. For tube formation assay, polymerized Matrigel (BD Biosciences, San Jose, CA, USA) was added to 48-well plates and HUVECs were seeded on it. Images were obtained after 12 h and the total branching points and total tube length were evaluated using ImageJ. Additionally, vasculogenesis-related gene expression, including vascular endothelial growth factor A *(VEGF-A)*, *VEGF-B* and fibroblast growth factor 2 *(FGF2),* was quantified by real-time PCR in HUVECs cultured with different scaffolds for 24 and 48 h [23], with primers as previously reported [24].

### In Vitro Osteogenesis-Related Gene Expression of hBMSCs

Human bone mesenchymal stem cells (hBMSCs) were originally purchased from Cell Bank of Shanghai Institutes for Biological Sciences. The mRNA expression of collagen 1 (*COL1*), Runt-related transcription factor 2 (*Runx2*), osteocalcin (*OCN*) and, osteopontin (*OPN*) were quantified to assess the osteogenic differentiation of different scaffolds using real-time PCR. Cells were adhered in 6-well plates and RNA were harvested after osteogenic induction for 7 days using TRIzol reagent (Invitrogen). PrimeScript RT reagent kit (Takara, Shiga, Japan) was used for mRNA to be reversely transcribed into complementary DNA. ABI 7900 was used for quantitative analysis of the reverse transcription reaction. The data were normalized to glyceraldehyde-3-phosphate dehydrogenase (*GAPDH*) expression and analyzed by the 2^−ΔΔCt^ method.

### Alizarin Red S Staining

Alizarin red S staining was used to evaluate the extracellular calcium deposition, revealing individual osteoinduction capacity of different scaffolds. HBMSCs were seeded into 24-transwell plates and co-cultured with BGS/NBGS in osteogenic differentiation medium for 21 days. After 21 days, cells were washed twice with PBS and fixed with 4% paraformaldehyde for 15 min and then stained with Alizarin Red S (2% aqueous, Sigma) solution for 30 min.

### In Vivo Photothermal Ablation Therapy

All animal procedures were approved by the Research Ethics Committee of Shanghai Sixth People’s Hospital. For in vivo photothermal therapy (PTT) evaluation, 1 × 10^5^ Saos-2 cells were injected subcutaneously into 4-week-old healthy and female nude BALB/c mice (body weight ≈ 16 g; Beijing Vital River Laboratory Animal Technology Co., Ltd.) to establish ectopic osteosarcoma model. 24 osteosarcoma-bearing mice were randomly divided into 4 groups (BGS, BGS + NIR, NBGS, NBGS + NIR; n = 6) when the tumor volume reached about 180 mm^3^. Small incisions around the edge tumor were made in the skin, and then, the capsule of the tumor was cut open. After BGS or NBGS scaffold (8 × 1.5 × 1.5 mm^3^) was implanted into the center of the tumor, the wound was closed. Twenty-four hours after BGS/NBGS implantation, NIR irradiation (1064 nm, 1.0 W cm^−2^, 5 min) was executed on the BGS + NIR and NBGS + NIR groups. A thermal imaging instrument (FLIRTM A325SC camera, USA) was used to collect the thermal images of the tumor site from all animals. The tumor tissues from one animal in each group were sectioned and stained with H&E, TUNEL and Ki-67 one day after the osteosarcoma ablation for histological analysis. Major organs were also sectioned and stained with H&E to observe in vivo toxicity. Survival time of the mice (*n* = 5) in different groups was recorded to draw survival curves.

### Surgical Models of Calvarial Defect and Scaffold Implantation

Twenty-four male Sprague–Dawley (SD) rats were used for surgical models of large calvarial defect and reparation with scaffold implantation. In detail, after anesthesia and sterilization, the skin was incised to expose the calvarial sagittal suture. Then, 25-mm-diameter defects were made in the frontal parietal bone using a slow speed electric trephine. The defects were implanted with BGS on the left side and NBGS on the contralateral side. Finally, the periosteum and skin were sutured separately to close the incision. Tetracycline hydrochloride, calcein AM and alizarin red were injected subcutaneously every 2 weeks after in vivo implantation of the scaffold. After another 3 weeks, half of the rats were executed and then perfused with Microfil (MV-112, Flow Tech, Inc., Carver, MA) after cardiac perfusion with heparinized saline and 4% paraformaldehyde solution. The rats were kept at 4 °C overnight, and then, calvarial specimens were obtained and fixed with 4% formalin. The rest of rats were successively executed by an overdose of anesthetic after 8, 16 and 24 weeks. Peripheral blood and major organs were obtained for respective analysis. Craniums were harvested and then treated with 4% paraformaldehyde solution for 24 h before further analysis.

### Micro-CT Scanning and Analysis

After fixed in 4% paraformaldehyde solution overnight, the newborn osseous regeneration surrounding the defect region were evaluated using the micro-CT-80 system (Skyscan, Kontich, Belgium). The specimens were scanned at a resolution of 18 μm per voxel according to the established protocol [15, 25]. After 3D reconstruction, bone mineral density (BMD), bone volume ratio (BV/TV) and total porosity (TOT) were calculated to assess the newborn osseous tissue using the auxiliary software of the mCT-80 system. After the scanning, half of the craniums were decalcified with a 10% EDTA solution. The samples were scanned again to reconstruct the vessels surrounding the calvarial defects using CTVol software after decalcification.

### Histopathological Staining

The rest craniums were dehydrated with graded ethanol and then embedded for histologic analysis to observe the newborn osseous tissue in the calvarial defects implanted with BGS/NBGS. The specimens were cut into 5-mm-thick sections and then incubated at 60 °C for 1.5 h after decalcification and paraffin. Hard tissue was sectioned to demonstrate newborn bone tissue and scaffold degradation in the defect using a CLSM. To evaluate the newborn osseous tissues, sections were stained with hematoxylin and eosin, Masson trichrome and Goldner trichrome solutions. Photomicrographs were acquired using a LEICA DM 4000. For Goldner trichrome staining, sections were kept in Weigert's hematoxylin for 30 min, washed in double-distilled water for 10 min and then stained with ponceau acid fuchsin, phosphomolybdic acid–orange G solution and light green stock solution sequentially.

### Data Analysis

Multivariate parametric data were analyzed using analysis of variance (ANOVA) with Tukey’s post hoc test. Statistical comparisons between two groups were based on Student's two-sided *t* test as ^*^p < 0.05, ^**^p < 0.01 (statistically significant). Quantitative data are reported as mean ± sd.

## Results and Discussion

### Synthesis and Characterization of 2D Nb_2_C MXene NSs

**Fig. 1:**
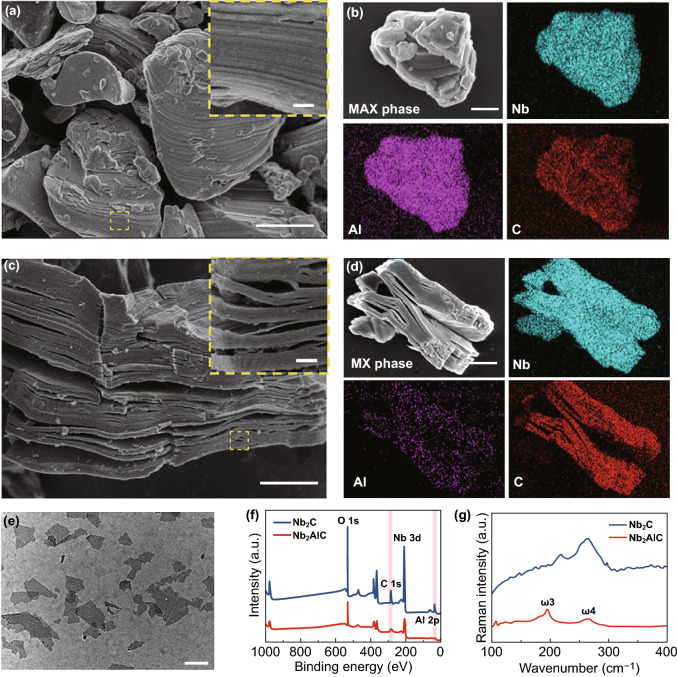
Fabrication and characterization of ultrathin 2D Nb_2_C MXene NSs. **a**, **b** SEM images of Nb_2_AlC ceramics with corresponding element mapping (Nb, Al and C). **c, d** SEM images of multilayered Nb_2_C MXene and the corresponding element mapping (Nb, Al and C). **e** TEM image of one-layered or few-layered Nb_2_C MXene NSs. **f** X-ray photoelectron spectroscopy (XPS) spectra of Nb_2_AlC bulk and Nb_2_C NSs. **g** Raman spectra of Nb_2_AlC bulk and Nb_2_C NSs. The scale bar in plane **a-c** equals 1 μm, and the bar of inset **a** and **c** represents 100 nm. The scale bar in plane **e** is 200 nm. (Color figure online)

Typically, 2D Nb_2_C MXene NSs were fabricated by a chemical exfoliation method, which involved selective HF etching and tetrapropylammonium hydroxide (TPAOH) intercalation [12]. SEM images revealed that the niobium aluminum carbide (Nb_2_AlC) ceramics (MAX phase), as prepared by solid-phase sintering, featured a densely layered microstructure (Fig. 1a). The SEM image and corresponding element content mapping exhibited that this MAX phase ceramic was ternary compounds including Nb, Al and C (Fig. 1b). When the MAX phase solid was treated with HF acid, the multilayered structure was formed (MXene, Fig. 1c, d) and the content of Al was decreased obviously (Fig. 1d). After further intercalated by TPAOH, the few-layered Nb_2_C NSs were obtained and observed by TEM (Fig. 1e), showing that Nb_2_C NSs were almost transparent with an ultrathin 2D structure. To further investigate the chemical status of Nb_2_AlC bulk and Nb_2_C NSs, XPS survey was operated (Fig. 1f). It was demonstrated that compared with Nb_2_AlC, the signal strength of Nb element in Nb_2_C NSs was significantly enhanced, while that of Al element was reduced. In agreement with XPS data, Raman spectra revealed the element Al exhibited a significant loss in Nb_2_C NSs than in Nb_2_AlC powder (Fig. 1g). After the treatment of HF and TPAOH, the typical vibration mode ω_3_ was weakened or even disappeared, which implied that the Al layer was largely removed. The vibration mode ω_4_ still existed, indicating the Nb_2_C NSs inherited the layered structure [26].

### Synthesis and Characterization of 3D NBGS

Consistent with our previous reports [14, 15], the as-prepared Nb_2_C NSs suspension presented a strong and broad UV–Vis–NIR absorption including the first biological window (NIR-I; 750–1000 nm) and the second spectral window (NIR-II; 1000–1700 nm), with the maximum absorbance of ~ 700–1200 nm. In particular, NIR-II biowindow has attracted more interest for its higher spatial resolution and the deeper light penetration compared to the traditional NIR-I biowindow [27, 28]. The previous studies [16] have demonstrated that the effective photothermal ablation based on NIR-II laser of subcutaneous xenograft tumor in nude mice could be achieved at the depth of ~ 4 mm. In addition, NIR-II laser achieved less attenuation of photothermal heating compared to NIR-I laser penetrated through the same depth of the tissues. It has been suggested that Nb_2_C NSs have high potential for antitumor as a desirable PTT agent. To endow the 3D printing BGS the PTT capacity, Nb_2_C NSs were coated onto the surface of BGS via capillarity [29]. In this study, 3D printing bone-mimetic BGS were directly soaked in different concentrations of Nb_2_C NSs aqueous solution to obtain Nb_2_C-coated BGS. BGS coated by 0.5 mg mL^−1^ Nb_2_C NSs were termed as 0.5 NBGS, and other NBGS were nominated by the same analogy.

**Fig. 2:**
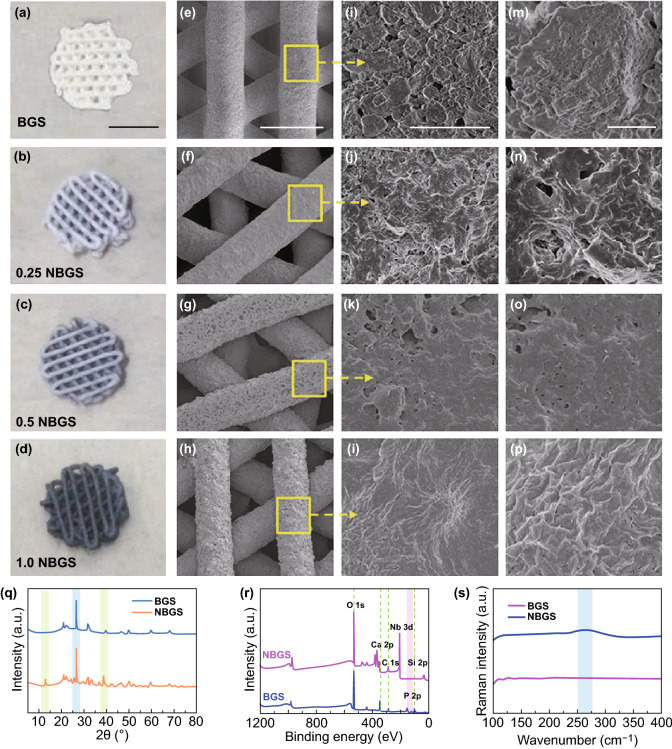
Synthesis and characterization of 3D NBGS. **a–d** Digital photographs of BGS and NBGS with 3D geometrical structure. The scale bar is 3 mm. **e**-**p** Photographs and different magnifications of SEM images of NBGS and BGS. From left to right, the scale bar is 500 μm, 5 μm and 1 μm, respectively. **q** XRD patterns of BGS and NBGS. **r** XPS spectra of BGS and NBGS. **s** Raman survey of BGS and NBGS. (Color figure online)

Intuitively, from BGS, 0.25 NBGS, 0.5 NBGS to 1.0 NBGS, the color of different scaffolds gradually changed from white to black (Figs. 2a–d), but their 3D well-defined microporous structures were not altered during Nb_2_C NSs modification (Figs. 2e–h). Additionally, with the concentration of Nb_2_C NSs elevated, Nb_2_C NSs on the surface of NBGS increased accordingly (Figs. 2i–l), while the micropores decreased (Figs. 2m–p). Further, cross-sectional morphologies and enlarged interfacial SEM images of NBGS demonstrated the successful integration of Nb_2_C NSs and BGS (Figs. S1e–g). The integration of NBGS was also validated by SEM-MAPPING on the surface of the composite scaffold, which revealed the co-localization of the two primary elements: Si from the BGS and Nb from Nb_2_C NSs (Figs. S1a–d). The corresponding elemental distribution tendencies of NBGS were detected by SEM–EDS (Fig. S2a). Distinctly, from core to shell, Nb element content increased, while Si and O elements content sharply decreased. XRD analysis (Fig. 2q), XPS survey (Figs. 2r and S2c–f) and Raman spectra of BGS and NBGS (Fig. 2s) also provided solid evidences that Nb_2_C NSs were successfully integrated onto the surface of BGS. Reflected by XRD, there is a new peak at 20° for NBGS, in accordance with Nb_2_C NSs (Fig. 2q). XPS peaks exhibited NBGS with an obvious peak of Nb 3d at ~ 200 eV binding energy compared with BGS (Fig. 2r). Raman curves showed NBGS with an additional peak at ~ 250 cm^−1^ (Fig. 2s), which is consistent with the ω_4_ vibration mode of Nb_2_C in Fig. 1g.

### Photothermal Property and Antitumor Performance of NBGS

Encouraged by the optical absorption property (Fig. S2e) and the previous demonstration that Nb_2_C NSs were promising as the NIR-II laser-activated photothermal conversion nanoagents [30], we explored the photothermal performance of the fabricated NBGS. As expected, NBGS induced a distinct temperature elevation when exposed to 1064-nm laser irradiation (Figs. S3a–d). The temperature was Nb_2_C concentration-dependent and laser power density dependent on both dry (air) and wet (PBS) environment. These data prompted us to explore the potential cytotoxicity and phototherapeutic effect on human osteosarcoma cell line of Saos-2.

**Fig. 3:**
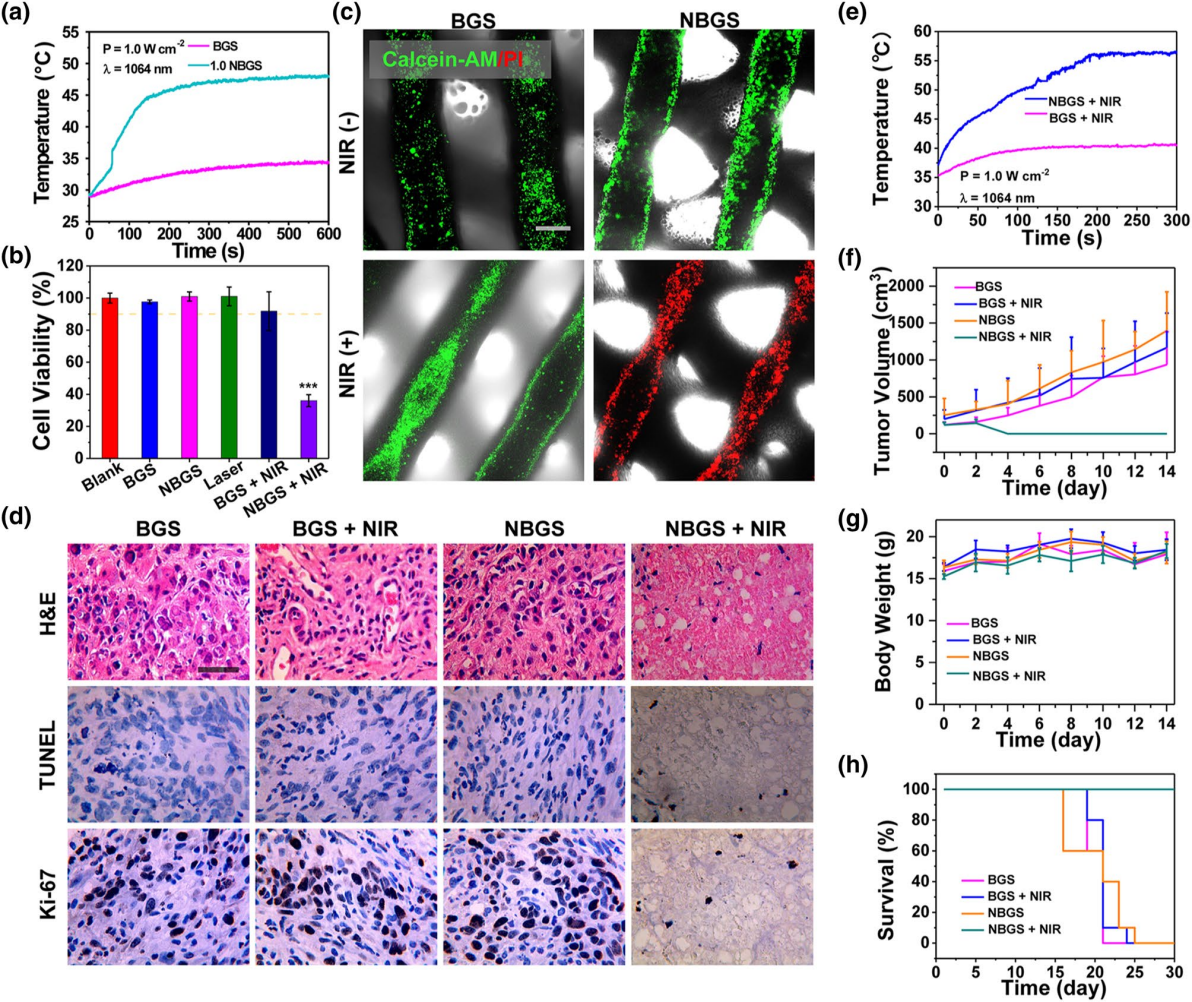
Photothermal property of BGS/NBGS and their antitumor capacities both in vitro and in vivo. **a** Temperature-change curves of 1.0 NBGS and BGS in PBS. **b** Relative cell viabilities of Saos-2 cells after various treatments. **c** CLSM images of all treatment groups (BGS, BGS + NIR, NBGS, NBGS + NIR) including bright-field images. Calcein AM/PI co-stained images indicated live/dead cells adhering on the scaffold. **d** Optical microscopy images of tumor tissues stained by hematoxylin and eosin (H&E), TdT-mediated dUTP nick-end labeling (TUNEL) and Ki-67. **e** Temperature curves of the tumor implanted with 1.0 NBGS and BGS in the mice model followed by NIR-II laser irradiation. **f** Tumor volume of osteosarcoma-bearing mice with different treatment protocols (n = 5). **g** Body weight of osteosarcoma-bearing mice (n = 5). **h** Survival curves of mice in different treatment groups (n = 5). The scale bar represents 500 mm (**c**) and 100 mm (**d**). (Color figure online)

Hence, cell viabilities in six groups (blank, BGS, NBGS, NIR only, BGS + NIR, NBGS + NIR group) were assessed by a standard CCK-8 assay (Figs. 3b and S3e–g). On the one hand, the cell viabilities in the blank, BGS and NBGS groups are above 90%, implying the low cytotoxicity and high biocompatibility of both BGS and NBGS. On the other hand, the NBGS + NIR group exhibited a higher cellular inhibition rate (> 62%), revealing highly efficient tumor cell ablation of NBGS as triggered by NIR laser. In addition, Annexin V-FITC/PI staining was used to assess the apoptosis of Saos-2 cells by flow cytometry (Fig. S4). The results demonstrated 50.6% apoptotic cancer cells in the NBGS + NIR-treated group, remarkably higher than that in other control groups (BGS, BGS + NIR, NBGS). Subsequently, Saos-2 cells incubated with BGS/NBGS were stained by calcein acetoxymethyl ester (calcein AM, green fluorescence) and propidium iodide (PI, red fluorescence) following NIR irradiation or not for further in vitro live/dead cells assay. As distinctly displayed in the CLSM images (Fig. 3c), the tumor cells in NBGS + NIR group presented conspicuously red signal, implying that the composite scaffold combined with NIR irradiation could effectively kill tumor cells. In a distinct contrast, the Saos-2 cells in BGS, BGS + NIR and NBGS groups revealed strong green florescence, demonstrating vital cells and rare cell death. The live/dead cell assay is completely in accordance with histological staining, CCK-8 data and flow cytometry assay.

Inspired by in vitro performance of killing osteosarcoma cells, in vivo antitumor experiment was performed to investigate the role of NBGS under NIR irradiation [31–34]. The phototriggered thermal ablation in NIR-II (1064-nm laser) was executed at the tumor site 24 h after the implantation of the scaffold. The temperature at the surface of xenograft was elevated to ~ 56 °C within 3 min of NIR irradiation, which was considerably higher than control groups (~ 40 °C) (Figs. 3e and S6a). The next day, the osteosarcoma ablation efficacies were investigated by H&E, TUNEL and Ki-67 staining on tumor specimen randomly harvested from each group (Fig. 3d). TUNEL and H&E staining exhibited that there were fewer apoptotic tumor cells in three control groups (BGS, BGS + NIR, NBGS). In contrast, apparent nuclear dissociation was observed in NBGS + NIR group, proving the death of Saos-2 cells. Ki-67 staining also supported the findings by TUNEL and H&E staining. These results manifested that NBGS were excellent antitumor agents, with high performance of NIR-II laser-triggered photothermal hyperthermia. Cytotoxicity of 2D materials and its complex interactions with systemic organs remain the issues of concern [35]. In order to evaluate the acute toxic effects of NBGS, the major organs of mice were harvested for H&E staining at 24 h post-laser irradiation. Obviously, there was no prominent inflammation infiltration or other pathological abnormalities in the treatment and control groups, implying the desirable biosafety of NBGS for photothermal bone tumor ablation (Fig. S6b).

After photonic therapy, the body weight and tumor volume of these mice were measured every two days, and the survival status was recorded simultaneously. NBGS plus laser irradiation effectively restrained the osteosarcoma growth, but protocols in control groups (BGS; BGS + NIR; NBGS) yielded ignorable therapeutic effects (Fig. 3f). The body weight of all animals displayed no significant difference within 14 days, suggesting the high biocompatibility and biosafety of NBGS (Fig. 3g). The survival time of animals in NBGS + NIR group was extended to 47 ± 4.5 days compared to 20 ± 2.1 days for BGS, 23 ± 1.3 days for BGS + NIR and 24 ± 2.2 days for NBGS group, suggesting that the tumor ablation by photothermal ablation could dramatically promote overall survival (Fig. 3h). These successful therapeutic outcomes confirm that NBGS have potentially clinical application as a highly efficient NIR-II-induced PTT agent with excellent biosafety both in vitro and in vivo.

### NBGS Stimulate Neovascularization both In Vitro and In Vivo

**Fig. 4:**
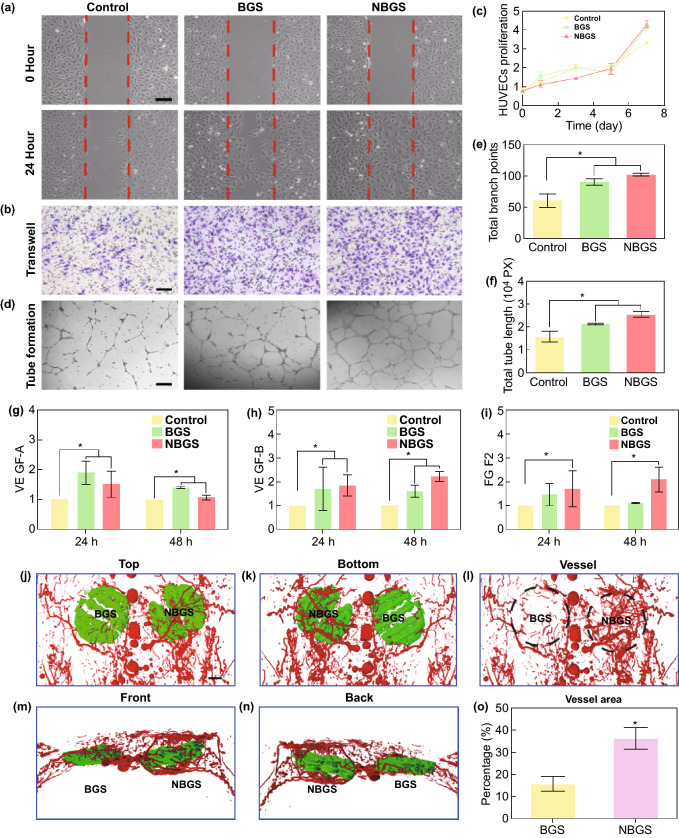
Neovascularization stimulated by BGS and NBGS in vitro and in vivo. **a** Wound-healing assay using HUVECs cultured with BGS and NBGS for 24 h. **b** Proliferation of HUVECs cocultured with BGS/NBGS for 1, 3, 5 and 7 days. **c** Representative photomicrographs of transwell migration assay of HUVECs after 24 h. **d** Tube formation of HUVECs stimulated by BGS and NBGS for 24 h. **e** Quantitative analysis of total branch points. **f** Quantitative analysis of total tube length. **g**–**i** Vasculogenesis-related gene expression (*VEGF-A*, *VEGF-B* and *FGF2*) in HUVECs cultured with different scaffolds after 24 and 48 h. **j–n** Reconstructed 3D micro-CT images of the blood vessels (red) surrounding the scaffolds (green) at 3 weeks. **o** Quantitative analysis of newborn blood vessels. The scale bar represents 250 μm (**a**–**c**) and 1 cm (**j**–**n**). (Color figure online)

Blood vessels play a crucial role in the entire process of skeletal development and postnatal bone repair [36, 37]. Neovascularization, including angiogenesis (i.e., formation of new capillaries from preexisting vessels) and vasculogenesis (i.e., de novo vessel formation) [38], has an intimate relationship with bone formation to repair the large cranial defect [39]. When bone injury and defect occur, triggered inflammation and recruitment of precursor cells through peripheral blood vessels are essential for the formation of newborn bone [40]. Because prior studies rarely showed the function of Nb element in new vasculature formation during bone regeneration [41, 42], we evaluated and compared the exact effects of BGS and NBGS on local vasculogenesis using human umbilical endothelial vein cells (HUVECs) model in vitro and vascular formation using microvascular perfusion and reconstruction in vivo. In general, the HUVECs model was used to assess the migration, proliferation and tube formation during vasculogenesis [43]. In detail, scratch wound healing assay (Fig. 4a) and transwell assay (Fig. 4b) were used to analyze the migration ability of endothelial cells co-cultured with BGS/NBGS. The results indicated that NBGS conspicuously accelerated the migration capacity of HUEVCs when compared with control and BGS group. The proliferation of HUEVCs was detected by CCK-8 protocol, showing no significant difference among three groups (Fig. 4c). Moreover, tube formation, presented as honeycomb-like structures to reveal the vasculogenic potential, showed an obvious priority when HUVECs were co-cultured with NBGS for 24 h (Fig. 4d). The capacity of tube formation was quantified by the branch numbers and tube length assay, which showed more branch numbers and elongated tubelike structures in NBGS group (Figs. 4e, f). In order to clarify the mechanism by which NBGS promotes a better angiogenesis, tube-related genes such as *VEGF-A*, *VEGF-B* and *FGF2* were detected by QPCR. As shown in Fig. 4g–i, both BGS and NBGS could upregulate vasculogenesis-related genes expression after continuous culture for 1 and 2 days. Nevertheless, the NBGS could persistently promote the expression of *VEGF-B* and *FGF2* when compared to BGS group, indicating a better performance to promote vasculogenesis. Taken together, these results demonstrated that the NBGS were non-cytotoxic to HUVECs and significantly strengthened vasculogenesis capacity of endothelial cells in vitro.

In vivo angiogenic performance surrounding the scaffold was explored through Microfil perfusion and angiography using micro-CT scanning after 3 weeks of implantation. Particularly, 3D micro-CT reconstruction (Figs. 4k–n) and quantitative analysis of newborn vessels (Fig. 4o) directly exhibited denser vascular networks encircling the NBGS. In vivo results showed an excellent angiogenic performance of NBGS, which is in line with in vitro findings*.* In addition, the blurry outlines of both scaffolds indicated their partial degradation. Therefore, the stimulatory vascularization by Nb released from NBGS, evidenced by both in vitro and in vivo findings, might become a desired companion for coupled osteogenesis, especially for the anatomical site with a poor circulation.

### NBGS Induce Osteogenesis In Vitro and Drive Bone Formation In Vivo

**Fig. 5:**
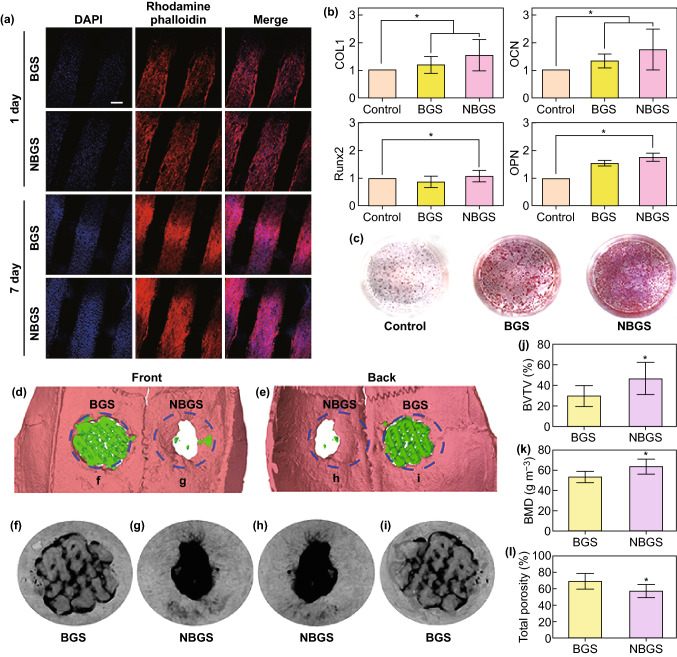
In vitro proliferation and osteogenic differentiation of hBMSCs and in vivo material-guided bone regeneration of BGS/NBGS. **a** Confocal fluorescence images of hBMSCs co-cultured with BGS/NBGS stained with DAPI (cell nuclei, blue fluorescence) and rhodamine phalloidin (cytoskeleton, red fluorescence) at day 1 and 7. The representative images show cellular proliferation and adherence on BGS/NBGS (scale bar, 200 μm). **b** Osteogenic genes expression (*COL1*, *RUNX2*, *OCN* and *OPN*) of hBMSCs in control, BGS and NBGS group at day 3. **c** Alizarin red staining of control, BGS and NBGS at the 21th day, showing mineralized extracellular matrix. **d, e** 3D reconstruction of micro-CT scanning was obtained to assess the defect and bone regeneration with different scaffolds. **f**–**i** Micro-CT analysis of harvested skulls collected from the experimental rats after 24 weeks. The morphology of undegraded BGS is distinct. **j**–**l** Bone regeneration capability as evaluated by quantitative analysis of fundamental parameters of two different scaffolds. (Color figure online)

Hydroxyapatite plays important roles in mesenchymal stem cells (MSC)’ proliferation and osteogenic differentiation. The Ca/P ratio which is closer to hydroxyapatite composition indicated a better mineralization capacity. Therefore, the potential of bioactive scaffolds to form apatite in SBF is of high significance [44, 45]. After immersed into SBF, the surface morphology of BGS changed from relatively smooth to rough with pronounced granular mineral deposition (Figs. S7a, b). The Ca/P ratio of the surface minerals was approximately 1.25 (Fig. S7c). Compared with BGS group, NBGS had more minerals deposited on the surface (Figs. S7d, e) and the Ca/P ratio of surface minerals (~ 1.53) was closer to the typical Ca/P of hydroxyapatite (= 1.67) (Fig. S7f). The Ca/P ratio of NBGS is beneficial for osteogenic differentiation and bone mineralization, yielding a better performance in bone regeneration in vivo. Cell–biomaterial interactions play important roles for biocompatibility and bioactivity [46–49]. Human bone marrow stem cells (hBMSCs) on NBGS and BGS attached tightly to the scaffolds, with well-spread morphology and many pseudopods penetrating into the 3D interconnected macropores of scaffolds (Figs. S7g, h), indicating high cytocompatibility of both scaffolds. Meanwhile, the proliferation and cytoskeleton staining of hBMSCs on NBGS and BGS were observed by CLSM (Fig. 5a). The cytoskeleton of hBMSCs was stained by rhodamine phalloidin and the nuclei were stained by DAPI (blue). The relatively stronger fluorescence at the 7th day than the 1st day showed the gradual propagation of hBMSCs. Notably, the NBGS induced better results of cell proliferation, larger cells spreading areas and morphological changes, revealing a better participation of Nb_2_C NSs for cell attachment, growth and proliferation.

Matrix mineralization is an essential process for the maturation of induced osteogenesis [50]. In the progress of osteogenesis and matrix maturation, a cascade of osteogenesis-associated biomarkers, including *RUNX2*, *OPN*, *COL1* and *OCN*, are upregulated. In our study, with hBMSCs cultured for 3 days, it was found that the expression of *COL1*, *OCN* and *OPN* in NBGS group were dramatically upregulated when compared with BGS group, reflecting an excellent bioactivity and osteoinductivity of NBGS (Fig. 5b). The alizarin red S staining was performed, as another indicator of extracellular mineralization. 24-transwell plates were used for alizarin red S staining with the scaffolds on the upper chamber co-cultured with hBMSCs for 3 weeks. The degree of extracellular calcium deposits in NBGS group was considerably more obvious than that of BGS and control groups (Fig. 5c), which demonstrated the promoted mineralization by NBGS. Therefore, the prepared NBGS, possessing excellent bioactivity, biocompatibility and osteoinductivity, could be used as an optimal biomaterial in driving bone regeneration.

The bone defect model in SD rats is the currently most common model for the research of bone defect regeneration. Very rare orthotopic osteosarcomatous model has been successfully established in the larger animals to evaluate the curative effect on tumor ablation and bone reconstruction simultaneously due to the intrinsic immunological rejection, which is the technical challenge and difficulty at current stage. Therefore, it is highly difficult to develop and establish osteosarcoma and bone defect in the same in vivo model. We established subcutaneous osteosarcoma model in nude mice to evaluate the photothermal effect of NBGS on tumor therapy and investigated the in vivo osteogenic capability of the NBGS in Sprague–Dawley rats. This strategy has been extensively adopted and accepted for evaluating the performances of bone tumor therapy and bone defect regeneration [14, 15, 51, 52]. In our experiments, we established a bone defect model on the both sides (right and left) of the crania of Sprague–Dawley rats, to implant NBGS and BGS into the defect site, respectively. The hyperthermia resulting from photothermal therapy may potentially damage the brain tissue next to the bone defect site. Therefore, the effect of NBGS combined with NIR-II laser on the outcomes of angiogenesis/neovascularization as well as osteogenesis, may not be recommended to be evaluated. Meanwhile, the previous related researches confirmed that the long-term bone regeneration process was not affected by the short-time NIR irradiation [12, 14]. The efficacy of NBGS in promoting calvarial regeneration was evaluated by micro-CT scanning (Figs. 5f–i). Bone regeneration capability of BGS and NBGS was evaluated by quantitative analysis of fundamental parameters based on the histomorphometric micro-CT analysis. (Figs. 5j–l). Bone volume/tissue volume (BV/TV) (Fig. 5j), bone mineral density (BMD) (Fig. 5k) and total porosity (TOT) (Fig. 5l) revealed the prominent role of Nb released from NBGS. BV/TV showed that the percentage of newborn bone tissue volume treated with NBGS group was significantly higher than the BGS group. The average BMD in the region of interest manifested the bone regeneration process from the circular defect areas, while the lower TOT indicated a better interior structure of newborn bone tissue [53].

Much more calcified tissue around the defect area in NBGS group confirmed the better efficiency for newborn bone regeneration than BGS group (Figs. 5d, e). Newborn osseous tissue of NBGS group almost filled up the defect area with few residual scaffolds left (Figs. 5f–i). The NBGS itself exhibited a desirable degradation rate and reconstruction rate of skeletal tissue, while more residual scaffold and fewer new osseous tissues were seen in BGS group. In addition, the peripheral blood was collected to investigate systemic toxicity of BGS/NBGS in vivo, and the major organs were obtained and sectioned for histological analysis after execution. Venous blood and organs from normal rats without any treatment were set as the control (Fig. S8). The results showed that both BGS and NBGS were featured with high biocompatibility, without significant pathological abnormalities in all animals.

**Fig. 6:**
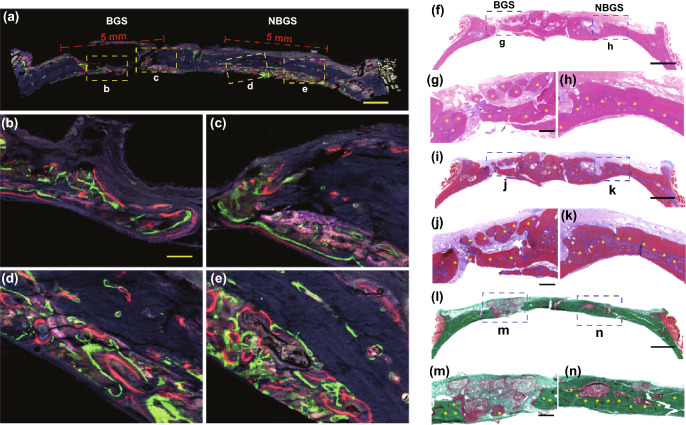
Fluorescent imaging and histological staining of the cranial bone defect to assess newborn osseous tissue. **a** Tetracycline hydrochloride (blue fluorescence), calcein AM (green fluorescence) and alizarin red (red fluorescence) (scale bar, 1 cm) were injected subcutaneously into rats with calvarial defect at week 2, 4 and 6, and different colors of fluorescence represent newborn bone tissue in different durations. Representative newborn bone induced by BGS **b**, **c** and NBGS **d**, **e** showing the new woven bone around the scaffold at week 8 (scale bar, 250 μm). **f**–**h** H&E staining, **i**–**k** Masson trichrome staining and **l**-**n** Goldner trichrome staining of the cranial defect implanted with BGS/NBGS at week 24. The scale bar equals 2 mm (**f**, **i**, **l**) and 200 μm (**g**, **h**, **j**, **k**, **m**, **n**). (Color figure online)

Confocal fluorescence images were conducted for further histological assessment. Different colors of newborn osseous represented dynamic bone formation (Figs. 6a–e). Blue fluorescence revealed the osteogenesis process during week 2–4, green fluorescence showed ossification from week 4 to 6 and red fluorescence reflected bone formation in the last 3 weeks. Compared with BGS, the newborn bone around NBGS exhibited excellent osteogenic performance (Figs. 6b–e). Green and red fluorescence in NBGS group was much more obvious than BGS group, indicating a better bone formation capacity during the last 5 weeks.

To evaluate the efficacy of NBGS and BGS for bone regeneration, a cascade of histological staining and analysis, including H&E staining (Figs. 6f–h), Masson trichrome staining (Figs. 6i–k) and Goldner trichrome staining (Figs. 6l–n), were further conducted to reveal microscopic reparation. A large amount of mineralized bone tissue (yellow arrows) was found in the bone defect implanted with NBGS as evidenced by H&E staining and Masson trichrome staining (Figs. 6g, h, j, k m and n) and no obvious residual scaffold left (Figs. 6g, j and m). Moreover, Goldner trichrome staining showed that the defect region in BGS group displayed a mixture of new osteoid tissue (red tissue) surrounding the residual materials (yellow arrows) (Fig. 6m). There was a great deal of mineralized bone tissue (emerald green tissue) filled in the defect region of NBGS group. The region of bone defect was fully filled with mineralized bone at week 24, without remnant NBGS scaffolds (Fig. 6n). This result showed the desirable capacities of bone regeneration and scaffold degradation.

**Fig. 7:**
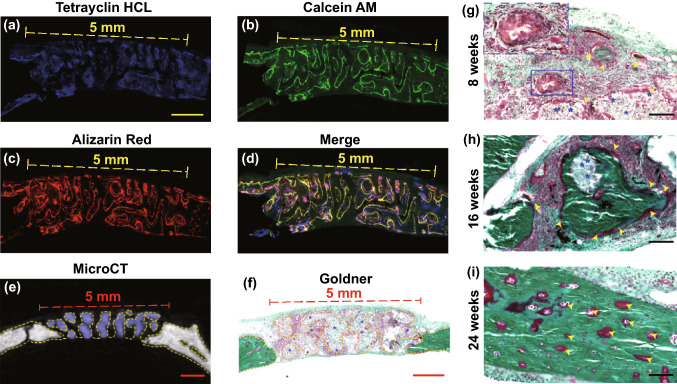
Material-guided bone regeneration of NBGS in vivo. **a**–**d** Confocal fluorescence images of the newborn osseous tissues in the circular defect regions were recorded at week 8. **e** Transverse view of micro-CT image of harvested craniums. Purple area marks the undegraded scaffold. Yellow dotted line marks the location of the new bone. **f** Goldner trichrome staining of NBGS group at week 8, with yellow dotted line indicating the area where newborn bone tissue grows. Blue asterisks mark the residual materials. **g–i** Goldner trichrome staining of the regenerated tissue in NBGS group at week 8, 16 and 24. Yellow arrow heads mark newborn osseous tissue. Blue asterisks mark the residual materials. The scale bar equals 1 mm (**a**–**f**) and 200 μm (**g**–**i**)

CLSM images of the newborn osseous tissues in the circular defect regions presented along the pores of the scaffold (Figs. 7a–d). Corresponding to these results, the yellow dotted line (Figs. 7e, f) along the space of the NBGS and the cranial surface marked newborn osseous tissue, indicating an excellent material-guided bone regeneration in vivo. Figure 7g–i shows newborn bone tissue formation of the NBGS during different periods (week 8, 16 and 24). Images at week 8 showed a myriad of fibroblasts distributed among the hierarchical space of scaffolds, similar to the early stage of bone formation. The osteoid in red color was gradually increased and interconnected around the biomaterials along with the scaffold biodegradation at week 16, demonstrating the fabulously synchronized processes of bone formation and scaffold degradation in NBGS group. The excellent therapeutic efficacy is attributed to the desirable connectivity of the porous scaffolds, with desired properties of conductivity and inductivity of vascularization, which is essential to promote bone regeneration and scaffold degradation [9, 54, 55]. Nb-based species released by the biodegradation of Nb_2_C MXene can obviously promote the blood vessel formation, gathering more immune cells around the defect site to accelerate the degradation of NBGS. Meanwhile, large amount of nutrient substance, oxygen and BMSCs were transported through newborn vessels to the bone defect site to promote bone regeneration. Moreover, the degradation of NBGS provides sufficient space for the bone remodeling. In addition, calcium (Ca^2+^) and phosphate (PO_4_^3−^) released during the degradation of the scaffold can promote the mineralization of new bone tissue. Compared to the NBGS group, rare new blood vessels and insufficient raw material supply in the control group significantly slowed down the degradation rate of BGS scaffold. These make the process of scaffold degradation and new bone formation coordinate with each other, and the new bone tissue immediately replaces the scaffold material to fill the bone defect.

All these experiments both in vitro and in vivo indicated that Nb_2_C NSs integrated with BGS were highly biocompatible, proangiogenic, osteoconductive and osteoinductive, which facilitate the repair of large bone defects, along with the photothermal therapy to ablate bone malignancy.

## Conclusions

In summary, we have constructed a multifunctional 3D bone-mimetic scaffolds, which holds high biocompatibility, intriguing photothermal property in NIR-II biowindow and specific performance of driving coupled angiogenesis and osteogenesis. Taking the advantage of photonic-responsive performance of 2D Nb_2_C NSs under NIR-II laser irradiation, NBGS acquires deeper tissue penetration during NIR-II-triggered photonic hyperthermia and benefits significantly to the therapy of osteosarcoma, prolonging the lifespan of tumor-bearing mice. Meanwhile, the introduction of Nb element in 2D Nb_2_C MXene enables NBGS with the capability of promoting angiogenesis, which substantially facilitates osseous regeneration to repair large bone defect. The interconnected capacities of vasculature formation and bone regeneration merit the rising concern in tissue engineering. This intriguing scaffold has markedly enriched the options of treating bone malignancy and defect. Therefore, it is envisioned that NBGS as promising multifunctional bioscaffolds hold high potential in bone tissue engineering and photonic-responsive therapeutic applications on combating osteosarcoma.

## Electronic supplementary material

Below is the link to the electronic supplementary material.

Supplementary file1 (PDF 5096 kb)
