# Correction to: Nb2C MXene-Functionalized Scaffolds Enables Osteosarcoma Phototherapy and Angiogenesis/Osteogenesis of Bone Defects

**DOI:** 10.1007/s40820-022-00805-9

**Published:** 2022-03-07

**Authors:** Junhui Yin, Shanshan Pan, Xiang Guo, Youshui Gao, Daoyu Zhu, Qianhao Yang, Junjie Gao, Changqing Zhang, Yu Chen

**Affiliations:** 1 Institute of Microsurgery On Extremities, Shanghai Jiao Tong University Affiliated Sixth People’s Hospital, Shanghai, 200233 People’s Republic of China; 2 School of Life Sciences, Shanghai University, Shanghai, 200444 People’s Republic of China; 3 State Key Laboratory of High Performance Ceramics and Superfine Microstructure, Shanghai Institute of Ceramics, Chinese Academy of Sciences, Shanghai, 200050 People’s Republic of China; 4 Department of Orthopaedic Surgery, Shanghai Jiao Tong University Affiliated Sixth People’s Hospital, Shanghai, 200233 People’s Republic of China; 5 Department of Orthopedics, The Second Affiliated Hospital, The Navy Medical University, Shanghai, 200003 People’s Republic of China

## Correction to: Nano-Micro Lett. (2021) 13:30 10.1007/s40820-020-00547-6

The original version of this article unfortunately contain some mistakes in figure. The authors found that the curves in Fig. 1f, g were missing.

The corrected version of Fig. 1 is given below:

**Fig. 1:**
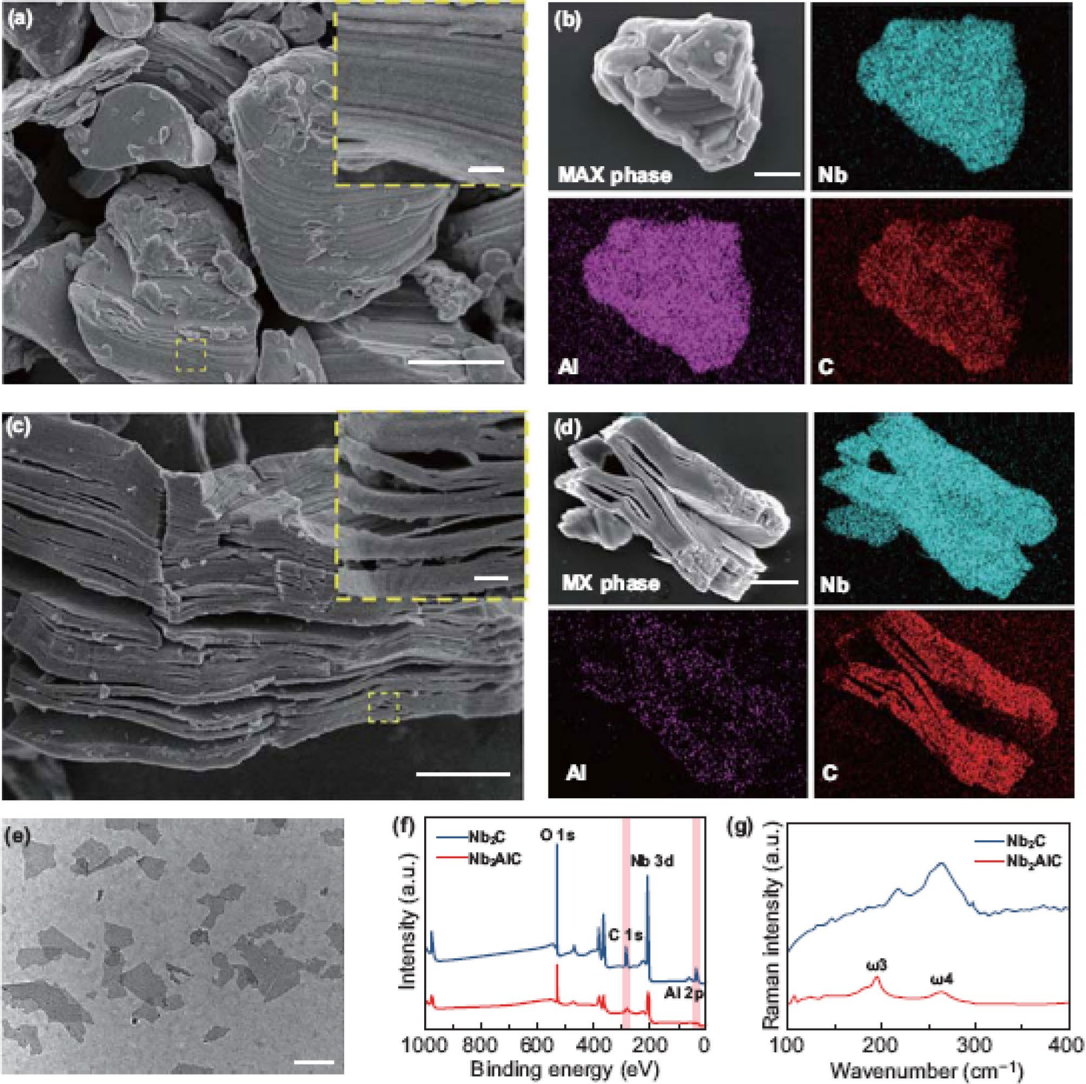
Fabrication and characterization of ultrathin 2D Nb_2_C MXene NSs. **a**, **b** SEM images of Nb_2_AlC ceramics with corresponding element mapping (Nb, Al and C). **c, d** SEM images of multilayered Nb_2_C MXene and the corresponding element mapping (Nb, Al and C). **e** TEM image of one-layered or few-layered Nb_2_C MXene NSs. **f** X-ray photoelectron spectroscopy (XPS) spectra of Nb_2_AlC bulk and Nb_2_C NSs. **g** Raman spectra of Nb_2_AlC bulk and Nb_2_C NSs. The scale bar in plane **a–c** equals 1 μm, and the bar of inset **a** and **c** represents 100 nm. The scale bar in plane **e** is 200 nm. (Color figure online)

