# Supplementary material for: Nb2C MXene-Functionalized Scaffolds Enables Osteosarcoma Phototherapy and Angiogenesis/Osteogenesis of Bone Defects
Source: Nanomicro Lett. 2021 Jan 4;13:30. doi: 10.1007/s40820-020-00547-6 (PMC8187678; doi:10.1007/s40820-020-00547-6)
Supplement: Supplementary file 1 — Supplementary file1 (PDF 5096 kb) [file 40820_2020_547_MOESM1_ESM.pdf]

Supporting Information for

Nb_2C MXene-Functionalized Scaffolds Enables Osteosarcoma Phototherapy and Angiogenesis/Osteogenesis of Bone Defects

Junhui Yin[1, 4, #], Shanshan Pan[2, 3, #], Xiang Guo[5], Youshui Gao[4], Daoyu Zhu[4], Qianhao Yang[4], Junjie Gao[4, *], Changqing Zhang[1, 4, *], Yu Chen[2, 3, *]

[1]Institute of Microsurgery on Extremities, Shanghai Jiao Tong University Affiliated Sixth People's Hospital, Shanghai 200233, People's Republic of China

[2]School of Life Sciences, Shanghai University, Shanghai 200444, People's Republic of China

[3]State Key Laboratory of High Performance Ceramics and Superfine Microstructure, Shanghai Institute of Ceramics, Chinese Academy of Sciences, Shanghai 200050, People's Republic of China

[4]Department of Orthopaedic Surgery, Shanghai Jiao Tong University Affiliated Sixth People's Hospital, Shanghai 200233, People's Republic of China

[5]Department of Orthopedics, The Second affiliated Hospital, The Navy Medical University, Shanghai 200003, People's Republic of China

[#]JunhuiYin and Shanshan Pan contribute equally to this work

*Corresponding authors. E-mail: colingjj@163.com (J. Gao); zhangcq@sjtu.edu.cn (C. Zhang); chenyuedu@shu.edu.cn (Y. Chen)

Supplementary Figures

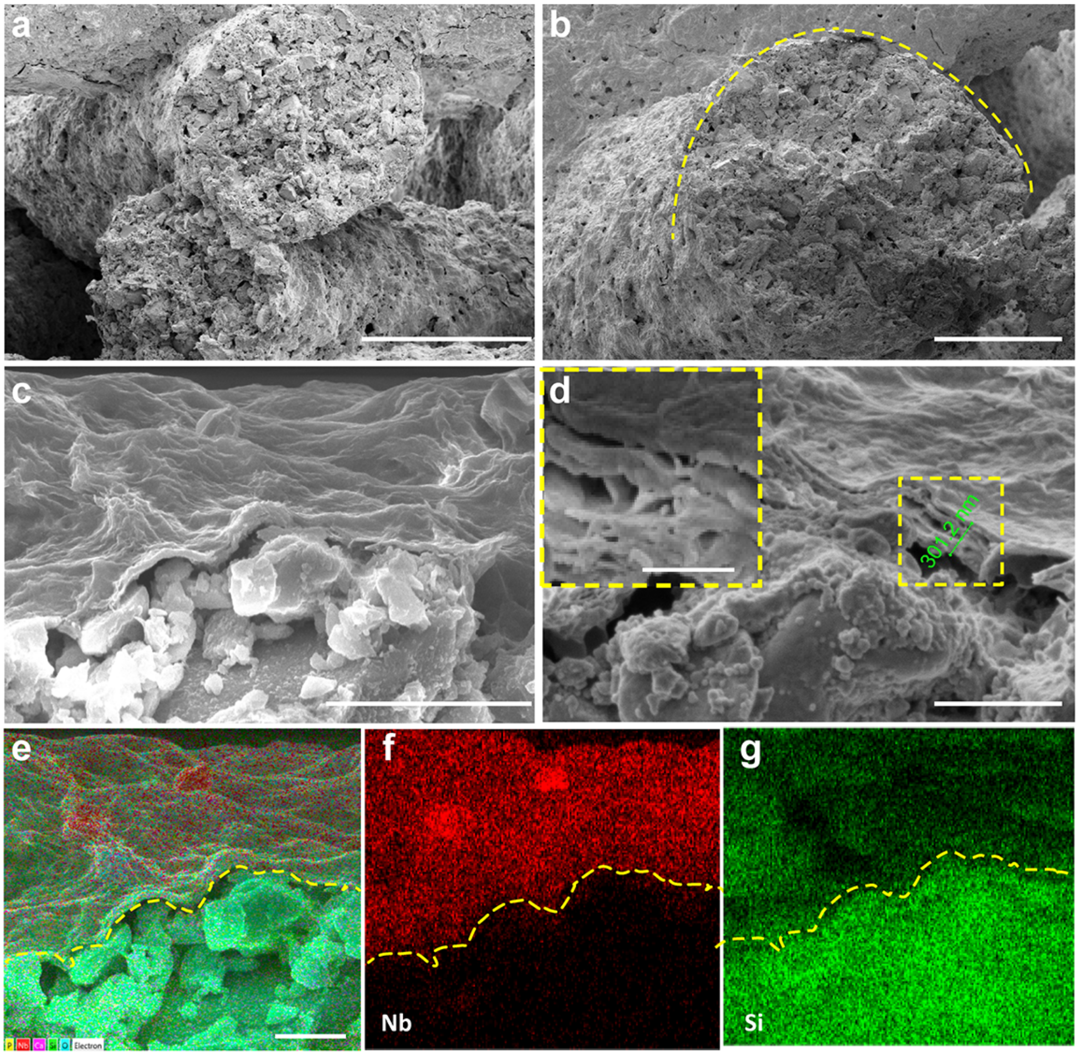

Fig. S1 a, b SEM images of cross section of 1.0 NBGS. Scale bars in plane **a** and **b** are 200 and 100 μm, respectively. **c, d** Enlarged interfacial SEM images in the presence of 2D Nb_2C MXene NSs shell (thickness ≈ 300 nm) and BGS core in 1.0 NBGS. Scale bar in plane **c**, **d** and the inset picture are 5 μm, 1 μm, and 100 nm, respectively. **e** Element distribution of fractured surface in NBGS. **f, g** Corresponding element mapping with e of Nb and Si in NBGS. All scale bars in plane **e-g** are 2 μm

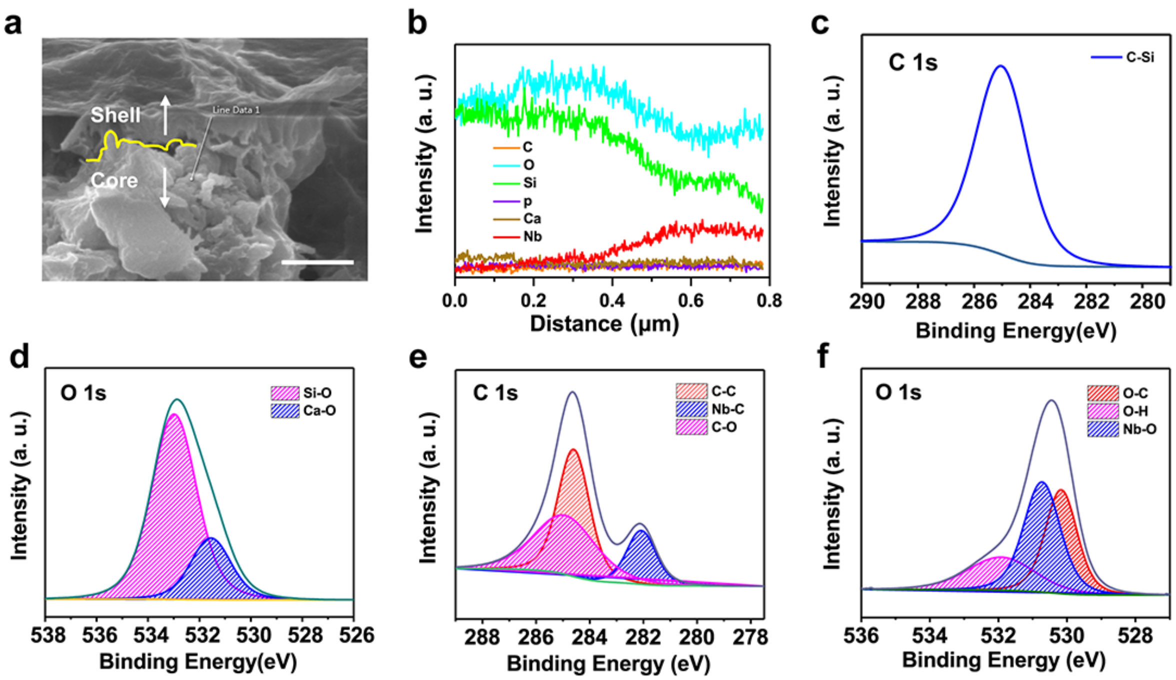

Fig. S2 a SEM image of core-shell structure in the cross section of 1.0 NBGS. Scale bar equals 1 μm. **b** Corresponding element distribution trend of NBGS in plane a. **c**, **d** XPS survey of C 1s and O 1s in BGS. **e**, **f** XPS survey of C 1s and O 1s in NBGS

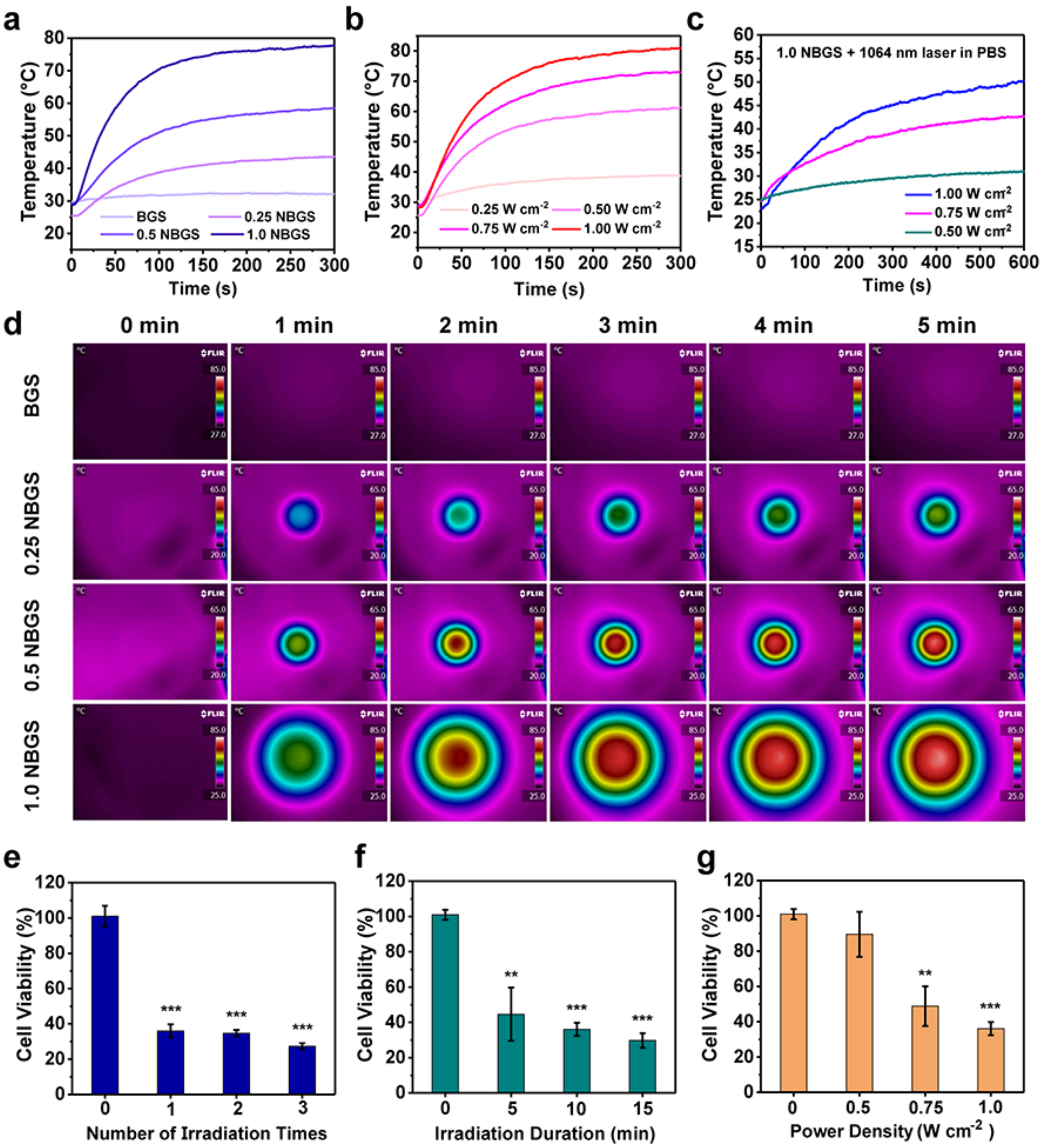

Fig. S3 a Temperature-change curves of BGS, 0.25 NBGS, 0.5 NBGS and 1.0 NBGS (1 W cm^{-2}, 5 min, in air). **b** Photothermal-heating curves of 1.0 NBGS under 1064 nm laser irradiation at the power densities of 0.25, 0.5, 0.75, and 1.0 W cm^{-2} (5 min, in air). **c** Temperature-change curves of 1.0 NBGS under 1064 nm laser irradiation at the power densities of 0.5, 0.75, and 1.0 W cm^{-2} (10 min, in PBS). **d** Photographs and IR thermal images of BGS, 0.25 NBGS, 0.5 NBGS and 1.0 NBGS under 1064 nm laser irradiation (1.0 W cm^{-2}, in air). **e** Relative cell viability of 1.0 NBGS under various irradiation durations (1.0 W cm^{-2}, 10 min, in 200 μL DMEM). **f** Relative cell viability of 1.0 NBGS under different irradiation durations (1.0 W cm^{-2}, in 200 μL DMEM). (**g**) Relative cell viability of 1.0 NBGS under gradient irradiation densities from 0 to 1.0 W cm^{-2} (10 min, in 200 μL DMEM)

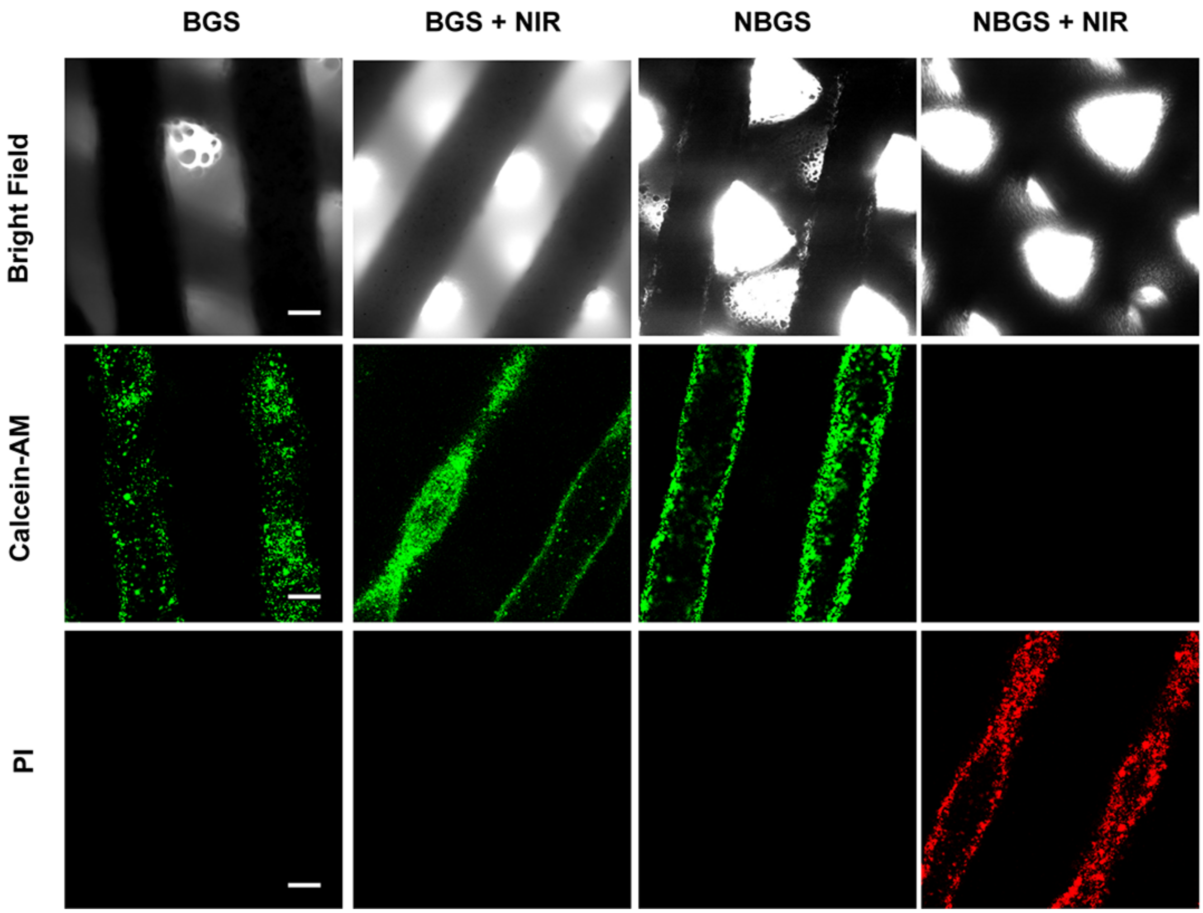

Fig. S4 CLSM images of BGS, BGS + NIR, NBGS and NBGS + NIR groups including bright field images, calcein-AM (living)/PI (dead) co-staining images. The scale bar is 200 μm. These images are the CLSM images in Fig. 3c with detailed information

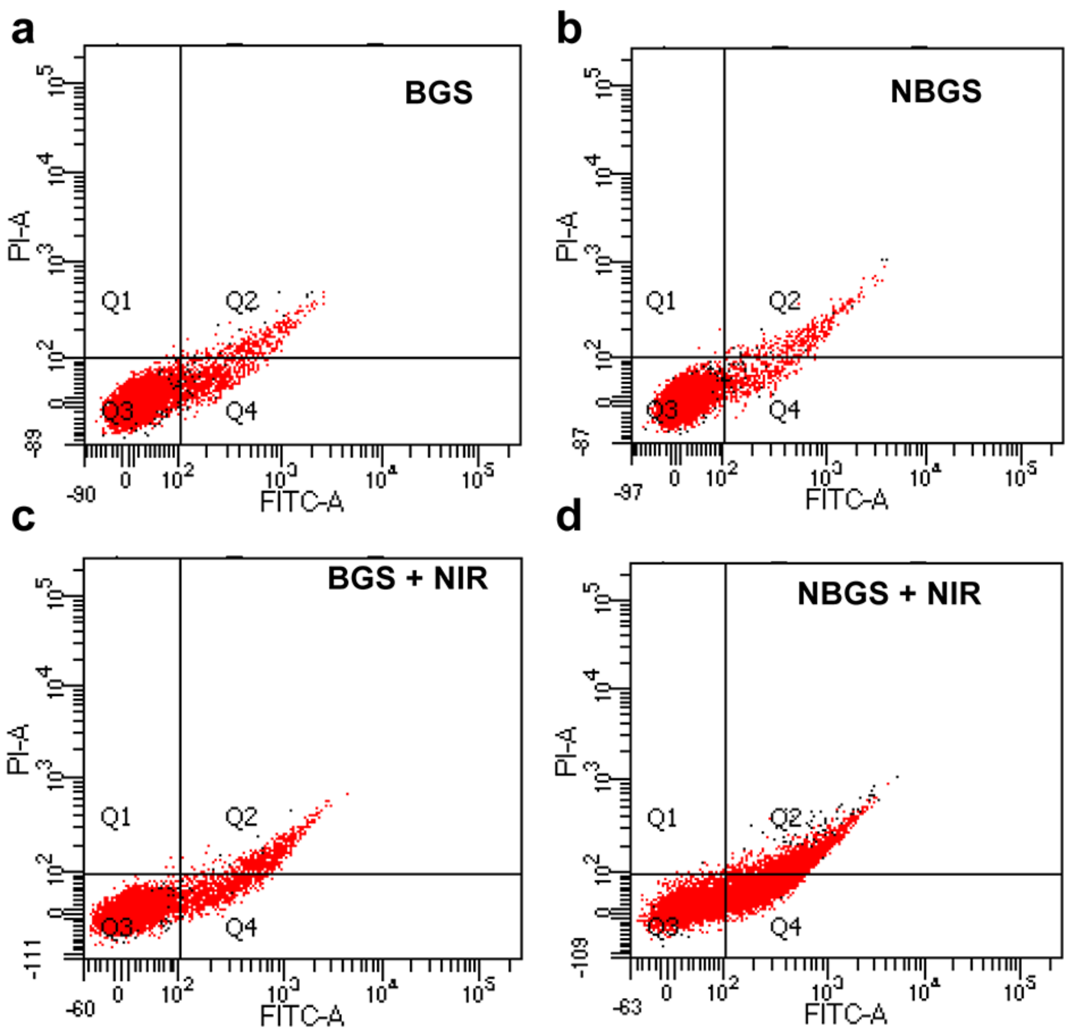

Fig. S5 Flow cytometry assay of Saos-2 cells on BGS/NBGS. Region Q1, Q2, Q3, and Q4 represents dead cells, late apoptotic cells, live cells and early apoptotic cells, respectively. **a** In BGS group, the percentages of cells in region Q1, Q2, Q3, and Q4 are 0.0%, 3.1%, 90%, and 6.8%, respectively. **b** In NBGS group, the percentages of cells in Q1, Q2, Q3, and Q4 are 0.0%, 3.3%, 92.8% and 3.9%, respectively. **c** In BGS + NIR group, cell distributions in area Q1, Q2, Q3, and Q4 are 0.0%, 5.0%, 87.2%, and 7.8%, respectively. **d** In NBGS + NIR group, the percentages are 0.2%, 16.3%, 49.2%, and 34.3% in Q1, Q2, Q3, and Q4, respectively

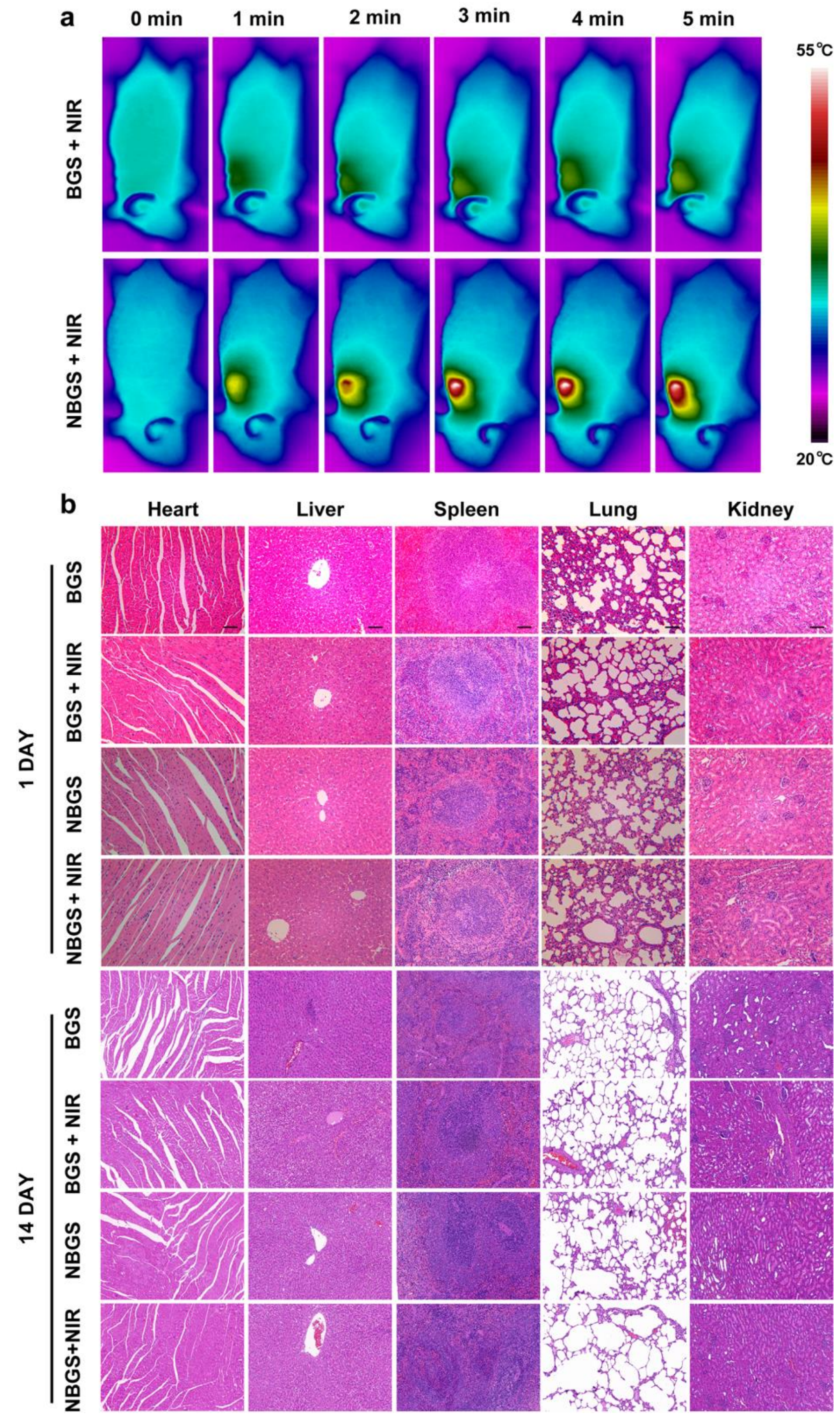

Fig. S6 a IR thermal images of tumor-bearing mice implanted with BGS and NBGS exposed to 1064 nm laser (1.0 W cm^{-2}, 5 min). **b** H&E staining of major organs at the 1th and 14th day after NIR laser irradiation. Scale bar represents 100 μm

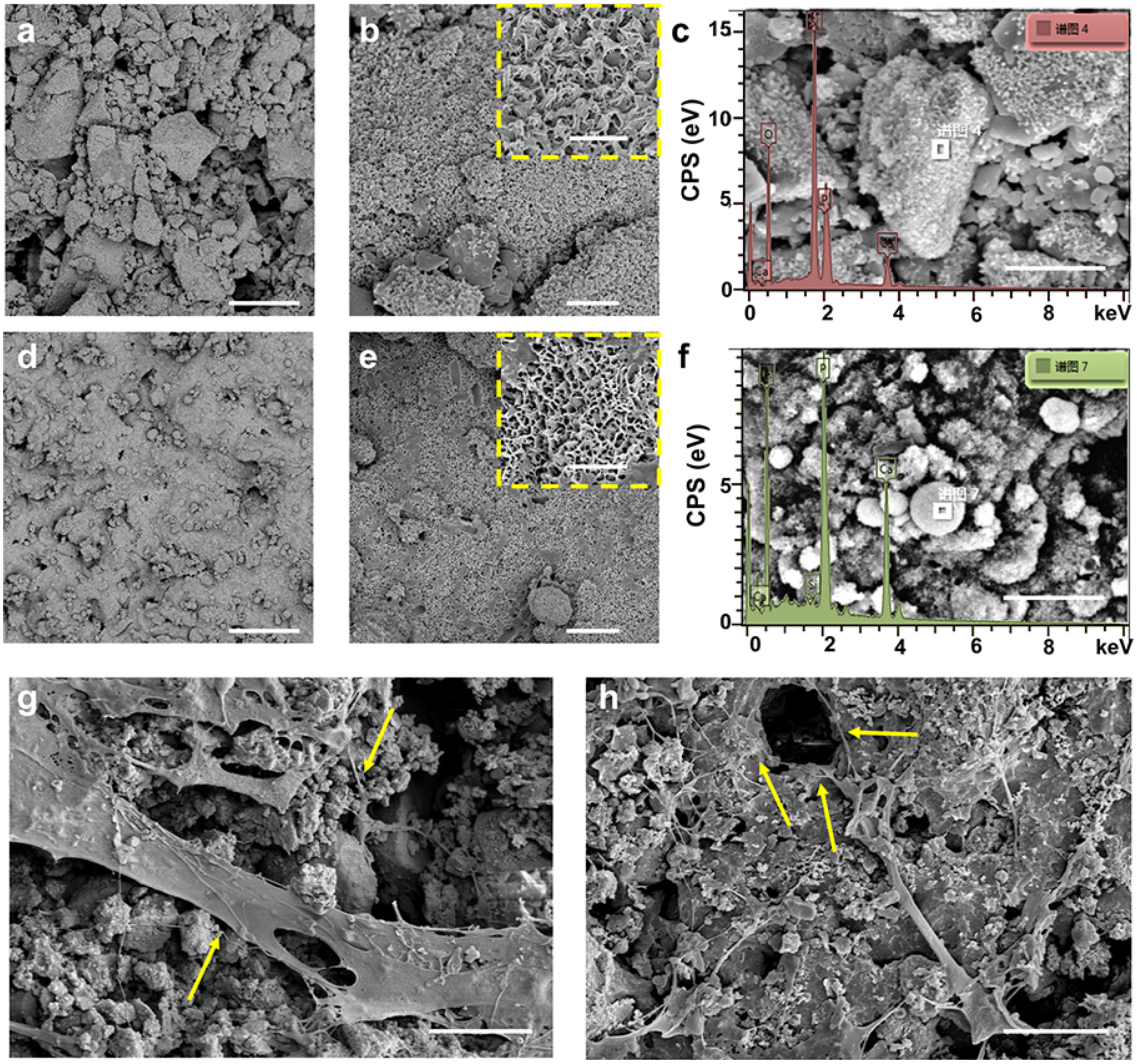

Fig. S7 a-c SEM images and EDS of BGS after being soaked in SBF for 1 day. The atomic contents of O, Si, P and Ca elements in the surface of BGS soaked in SBF are 59.84%, 27.61%, 5.58%, and 6.97%, respectively, and the Ca/P ratio approximately equals 1.25. The scale bar in plane **a, b, c** and inset is 20, 3, 5, and 1 μm, respectively. **d-f** SEM images and EDS of NBGS after soaked in SBF for 1 day. The atomic contents of O, Si, P and Ca elements in the surface of BGS soaked in SBF are 69.88%, 0.40%, 11.77%, and 17.95%, respectively. The ratio of Ca/P approximately equals 1.53. Scale bar in plane **d, e, f** and inset is 20, 3, 5, and 1 μm, respectively. **g** SEM image of hBMSCs in BGS after co-incubated for 1 day. Yellow arrows indicate extended pseudopods penetrating into 3D interconnected macropores of scaffolds. **h** SEM image of hBMSCs in NBGS after co-incubated for 1 day. Yellow arrows point out pseudopods. Scale bar in plane **g** and **h** is 5 μm.

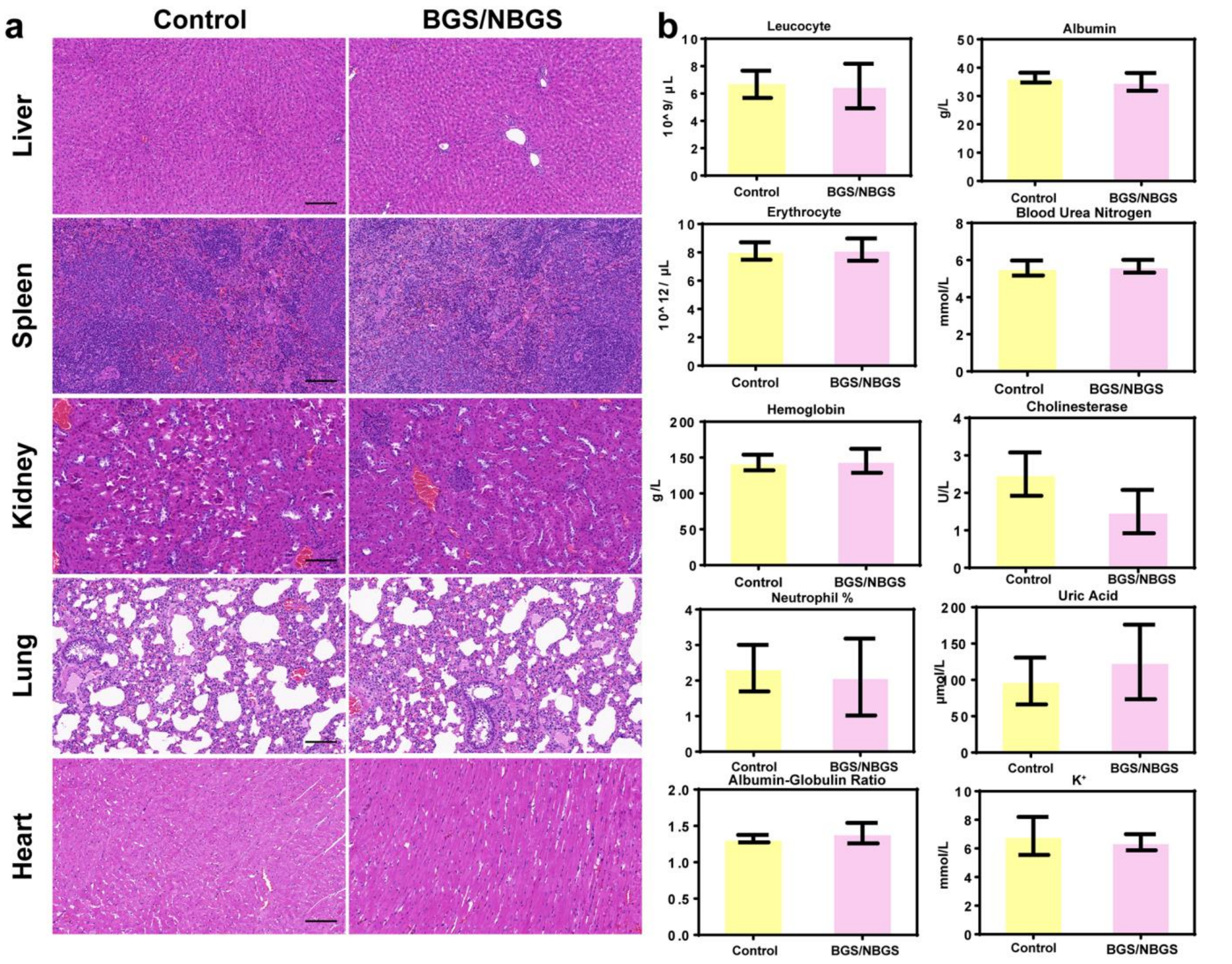

Fig. S8 a H&E staining of major organs from pristine animals (control) and those with BGS/NBGS implantation. There were no significant differences between the two groups. **b** Blood cell count and biochemical analysis of peripheral blood from SD rats with large calvarial defect and BGS/NBGS implantation. The results were compared to the control group. The scale bar represents 100 µm.

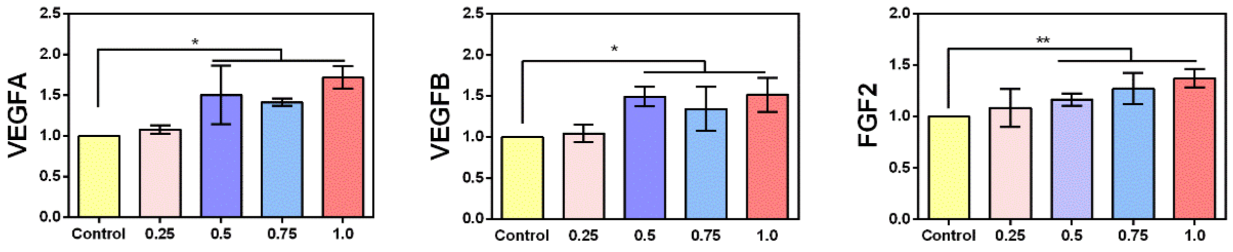

Fig. S9 Vasculogenesis-related gene expression (*VEGF-A*, *VEGF-B*, and *FGF2*) in HUVECs cultured with different concentration Nb_2C nanosheets (0, 0.25, 0.5, 0.75, and 1 mg mL^{-1}) after 24 h (*p < 0.05, **p < 0.01, statistically significant)
